# Increased Extracellular ATP: An Omen of Bacterial RTX Toxin-Induced Hemolysis?

**DOI:** 10.3390/toxins6082432

**Published:** 2014-08-15

**Authors:** Yifei Wang, Shijun Wang

**Affiliations:** 1 The East Chapel Hill High School, Chapel Hill, NC 27514, USA; 2 The Division of Molecular Pharmaceutics, UNC Eshelman School of Pharmacy, University of North Carolina, Chapel Hill, NC 27599, USA; E-Mail: yifei@email.unc.edu; 3 The Department of Pathology, School of Medicine, University of Washington, Seattle, WA 98104, USA

Bacterial infection is a major threat to human health. Although pathogenic bacteria vary in their virulence, it has been recognized that many pathogenic bacteria share common mechanisms when attacking host cells and tissues. Some pathogenic bacteria synthesize and secrete polysaccharides to form an extracellular capsule. Capsules serve as virulence determinants by multiple mechanisms including facilitation of bacterial adherence, evasion of the immune response, and antibiotic resistance [1]. Moreover, to the exterior of bacterial plasma membranes are certain toxic components (e.g., lipopolysaccharide (LPS) in Gram-negative bacteria, and peptidoglycan fragments and teichoic acids in Gram-positive bacteria) that play key roles in causing bacterial septic shock or multiple organ dysfunction [2]. Significantly, bacteria may secrete proteinaceous or non-proteinaceous molecules, namely exotoxins, capable of directly destroying host cells. The Repeat-in-Toxin (RTX) family is a group of virulence-associated exotoxins that are generated by Gram-negative bacteria and are noted for their ability to form pores on the membrane of host cells including leukocytes [3]. Despite the intense effort that has been input into investigating the interaction between RTX toxins and host cells during bacterial infection, our understanding of how RTX toxins insert into host cell membranes, and in turn, how host cells respond to the challenge of these toxins remains very limited. 

α-Hemolysin (Hly A) and leukotoxin A (Ltx A) are two typical RTX toxins that are secreted by bacteria *E. coli* and *Aggregatibacter actinomycetem*, respectively. Exposure of blood cells such as erythrocytes to these toxins may damage the integrity of cell membranes and eventually lead to cell death, a response that is called hemolysis. It is assumed that the interaction of these toxins with the host erythrocyte cell membranes is a two-step process, starting from a reversible absorption of toxins, followed by an irreversible toxin membrane insertion [4]. In most cases, host cells do not necessarily die immediately upon stimulation of toxins. Instead, they respond to the membrane insertion of RTX toxins like Hly A by prompting a calcium influx into cytosol, which is frequently accompanied with an increased release of cellular metabolites such as ATP to the extracellular space. Leakage of ATP may activate the P2X receptor, accelerating the Hly A-induced cytolysis [5].

The potential roles of extracellular ATP in Hly A-induced cytolysis raise questions on when and how ATP is released from erythrocytes stimulated by the RTX toxins Hly A and Ltx A. In a recent study, Skals *et al.* reported a rapid release of ATP from either Hly A or Ltx A-treated human erythrocytes, and suggested that the efflux of ATP was non-cytolytic in nature since it occurred before any signs of hemolysis. Using transgenic mice deficient in pannexin 1 and specific inhibitors to block those classical ATP-release pathways, including pannexin 1 and the voltage dependent anion channels, they excluded the possibility that the instant release of ATP was dependent on the transitional channels or transporters, and instead claimed that ATP was released through the RTX toxin-formed pores on the erythrocyte membranes [6]. 

The work by Skals *et al.* broadens our understanding of how RTX toxins cause hemolysis, especially confirming that an increased ATP efflux appeared earlier than hemolysis. In addition, they provided evidence that there must be a correlation between the toxin-host cell membrane interaction and the ATP release. However, such a correlation is not sufficient to answer the question of how RTX toxin-induced ATP efflux passed across cell membranes. Their results could be explained by the membrane instability that was inflicted by the presence of the RTX toxins. Clearly this is just a speculation but not a conclusion. Although they acknowledged that it is not established that the toxins forms pores, however, their conclusive title that “Bacterial RTX toxins allow acute ATP release from human erythrocytes directly through the toxin pore” leads to some questions because they had no direct evidence in support of such a claim. As acknowledged by the authors, whether pores really exist on the cell membranes of either Hly A or Ltx A-treated erythrocytes remains unproven. Although it is assumed that the size of Hly Atoxin-formed pores could be approximately 2 to 3 nm in diameter, this opinion is based on some experimental data collected by using indirect osmotic protection and electrophysiological techniques. In fact, electron microscopy, crystal structure analysis or other techniques have failed to directly reveal the pores formed by HlyA [7].

Extracellular ATP may either function as a signaling molecule regulating some physiological events such as pre-chondrogenic condensation [8] or play a pro-inflammatory role in LPS-induced inflammation [9]. Although further investigation into how ATP leaks out rapidly upon stimulation of the RTX toxins Hly A and Ltx A is essential, an instantly increased level of extracellular ATP usually predicts the ensuing hemolysis. Therefore, dynamically monitoring changes in the extracellular concentration of ATP and its metabolic products like ADP might provide a warning sign of hemolysis during bacterial infection.
